# Supplementary material for: Hybrid Models and Biological Model Reduction with PyDSTool
Source: PLoS Comput Biol. 2012 Aug 9;8(8):e1002628. doi: 10.1371/journal.pcbi.1002628 (PMC3415397; doi:10.1371/journal.pcbi.1002628)
Supplement: Text S4 — Complete source code for the PyDSTool package (version 0.88.120504). Includes API documentation and help files linking to web pages. This file is identical to the current public release on Sourceforge.net. (ZIP) [file pcbi.1002628.s004.zip › PyDSTool/html/PyDSTool.PyCont.Continuation.UserDefinedCurve-class.html]

xml version="1.0" encoding="ascii"?


PyDSTool.PyCont.Continuation.UserDefinedCurve


| Home | Trees | Indices | Help | PyDSTool |
| --- | --- | --- | --- | --- |

Package PyDSTool ::
Package PyCont ::
Module Continuation ::
Class UserDefinedCurve

[hide private]

[frames] | no frames]

# Class UserDefinedCurve

source code

```
  object --+    
           |    
Continuation --+
               |
              UserDefinedCurve
```

---

User defined curve. We'll see how this goes...


|  |  |
| --- | --- |
| Instance Methods | [hide private] |

|  |  |
| --- | --- |
| \_\_init\_\_(self, model, gen, automod, plot, initargs=None)  x.\_\_init\_\_(...) initializes x; see x.\_\_class\_\_.\_\_doc\_\_ for signature | source code |

|  |  |
| --- | --- |
| update(self, args)  Update parameters for UserDefinedCurve. | source code |

|  |  |
| --- | --- |
| \_createTestFuncs(self)  Creates processors and test functions for UserDefinedCurve class. | source code |

**Inherited from `Continuation`**:
`__copy__`,
`__deepcopy__`,
`__repr__`,
`__str__`,
`backward`,
`cleanLabels`,
`computeEigen`,
`display`,
`exportGeomview`,
`forward`,
`getSpecialPoint`,
`info`,
`reset`,
`testdomain`,
`testdomaingrid`,
`testdomaintangrid`

**Inherited from `Continuation`** (private):
`_Keller`,
`_MoorePenrose`,
`_Natural`,
`_checkForBifPoints`,
`_compute`,
`_curveToPointset`,
`_preTestFunc`,
`_savePointInfo`,
`_system`,
`_systemjac`,
`_systemjac_withpars`,
`_systemjacuser`,
`_systemuser`

**Inherited from `object`**:
`__delattr__`,
`__getattribute__`,
`__hash__`,
`__new__`,
`__reduce__`,
`__reduce_ex__`,
`__setattr__`


|  |  |
| --- | --- |
| Properties | [hide private] |

**Inherited from `object`**:
`__class__`


|  |  |
| --- | --- |
| Method Details | [hide private] |

|  |  |
| --- | --- |
| \_\_init\_\_(self, model, gen, automod, plot, initargs=None)  *(Constructor)* | source code |

x.\_\_init\_\_(...) initializes x; see x.\_\_class\_\_.\_\_doc\_\_ for
signature

Overrides: object.\_\_init\_\_: *(inherited documentation)*

|  |  |
| --- | --- |
| update(self, args) | source code |

Update parameters for UserDefinedCurve.

Overrides: Continuation.update

|  |  |
| --- | --- |
| \_createTestFuncs(self) | source code |

```
Creates processors and test functions for UserDefinedCurve class.

Note:  In the following list, processors are in PyCont.Bifpoint
and test functions are in PyCont.TestFunc.

Point type (Processor): Test function(s)
----------------------------------------

LP (FoldPoint): Fold_Tan
                Fold_Det
                Fold_Bor
H (HopfPoint): Hopf_Det
               Hopf_Bor
```

Overrides: Continuation.\_createTestFuncs

  


| Home | Trees | Indices | Help | PyDSTool |
| --- | --- | --- | --- | --- |

|  |  |
| --- | --- |
| Generated by Epydoc 3.0.1 on Fri May 4 15:24:07 2012 | http://epydoc.sourceforge.net |
